# Supplementary material for: The transcriptional repressor Blimp1/PRDM1 regulates the maternal decidual response in mice
Source: Nat Commun. 2020 Jun 3;11:2782. doi: 10.1038/s41467-020-16603-z (PMC7270082; doi:10.1038/s41467-020-16603-z)
Supplement: Supplementary file 1 — Supplementary Information [file 41467_2020_16603_MOESM1_ESM.pdf]

Supplementary Information

The transcriptional repressor Blimp1/PRDM1 regulates the maternal decidual response in mice

Mubeen Goolam, Maria-Eleni Xypolita, Ita Costello, John P. Lydon, Francesco J. DeMayo, Elizabeth K. Bikoff, Elizabeth J. Robertson and Arne W. Mould

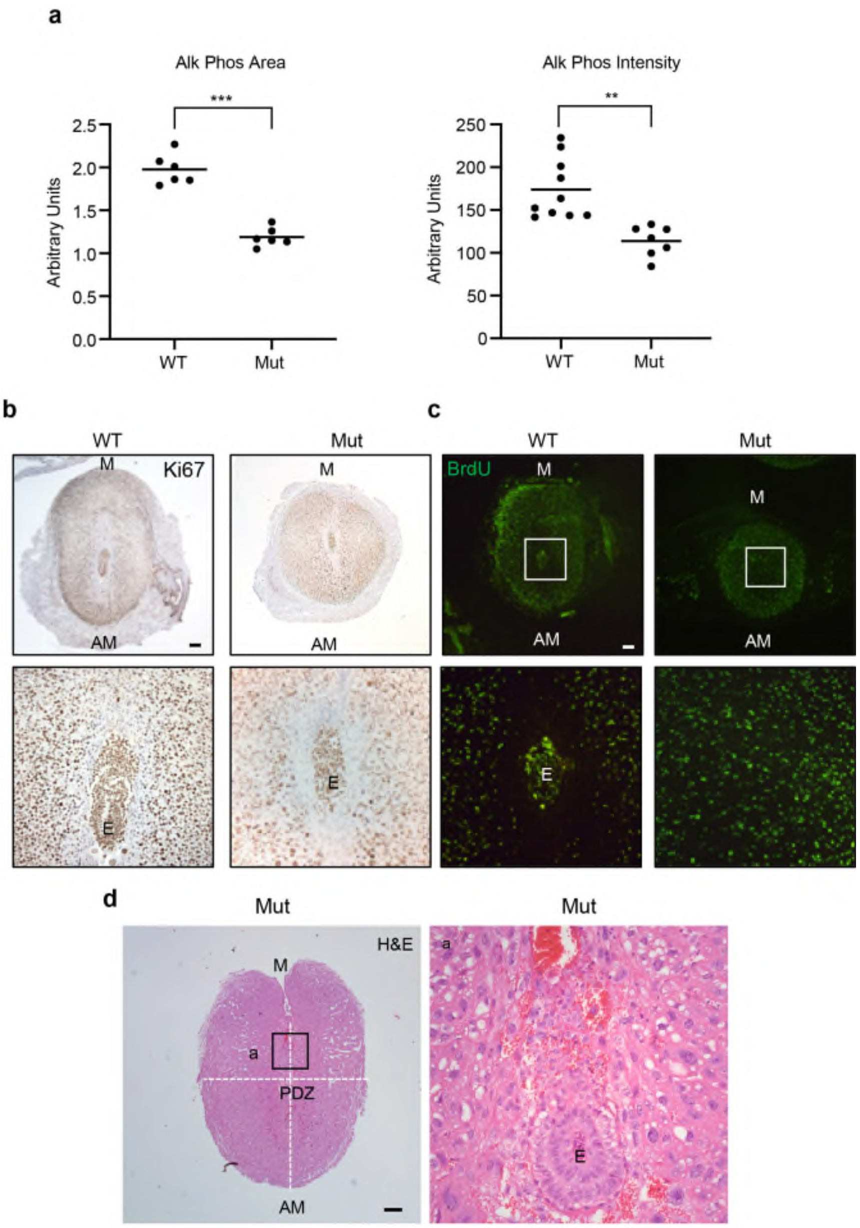

Supplementary Fig. 1. Reduced alkaline phosphatase but not proliferation in Blimp1 mutant decidua. Related to Fig. 2. **a**) Measurement of alkaline phosphatase staining area and intensity in E5.5 decidua confirms significant reductions in both parameters in Blimp1 mutants. Individual data points from analysis of 6-10 tissues sections from 3 independent decidual samples per genotype are shown. Bars represent mean. Two-tailed unpaired Student's t-test $^{**}p = 8.98 \times 10^{-4}$, $^{***}p = 3.45 \times 10^{-6}$. Source data are provided as a Source Data file. **b**) Ki67 IHC staining of E6.5 decidua indicates that proliferative capacity is unaffected in Blimp1 mutant mice. **c**) IF detection of BrdU incorporation in E5.5 decidua counterstained with DAPI confirms equivalent proliferative capacity between mutant and wild type decidua. **d**) H&E staining of E6.5 Blimp1 mutant decidua with myometrium removed showing disrupted embryo morphology and mislocalised implantation in the mesometrial region. PDZ = primary decidual zone, M = mesometrial, AM = antimesometrial, E = embryo. Scale bars = 100μm. All staining experiments were performed using a minimum of triplicate independent samples.

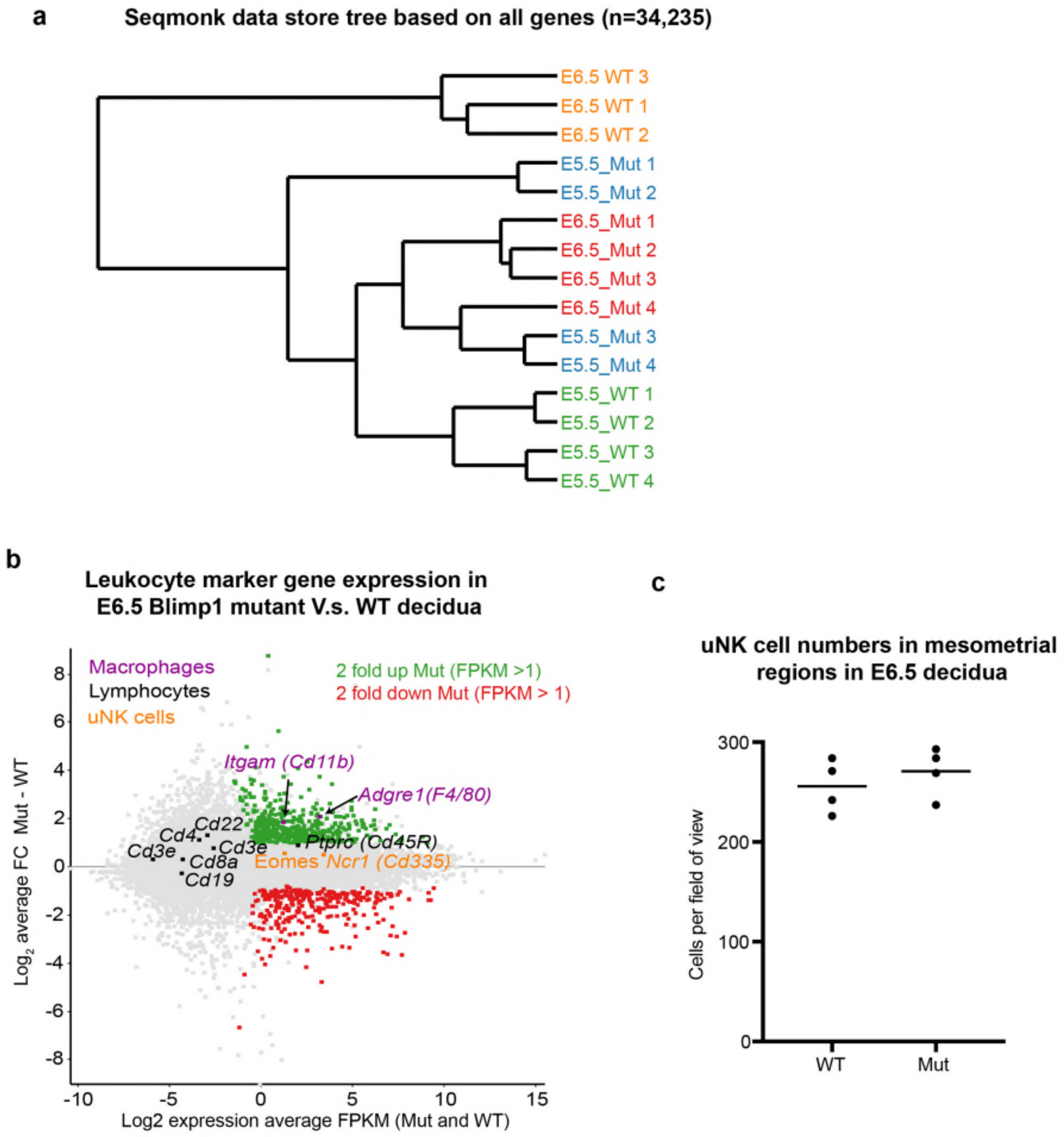

Supplementary Fig. 2. RNA-Seq analysis and uNK cell quantification. Related to Fig. 4. **a**) Hierarchical clustering of wild type and Blimp1 mutant decidual RNA-Seq samples based on expression of all genes. E5.5 and E6.5 Blimp1 mutants cluster with E5.5 wild type samples indicative of a stall in decidual development in mutants. **b**) RNA-Seq analysis of leukocyte marker expression in E6.5 mutant decidua suggests a selective increase in macrophages. **c**) Quantification of number of Eomes positive uNK cells in mesometrial region of wild type and Blimp1 mutant decidua. Individual data points from analysis of 4 independent decidual samples per genotype are shown. Bars represent mean.

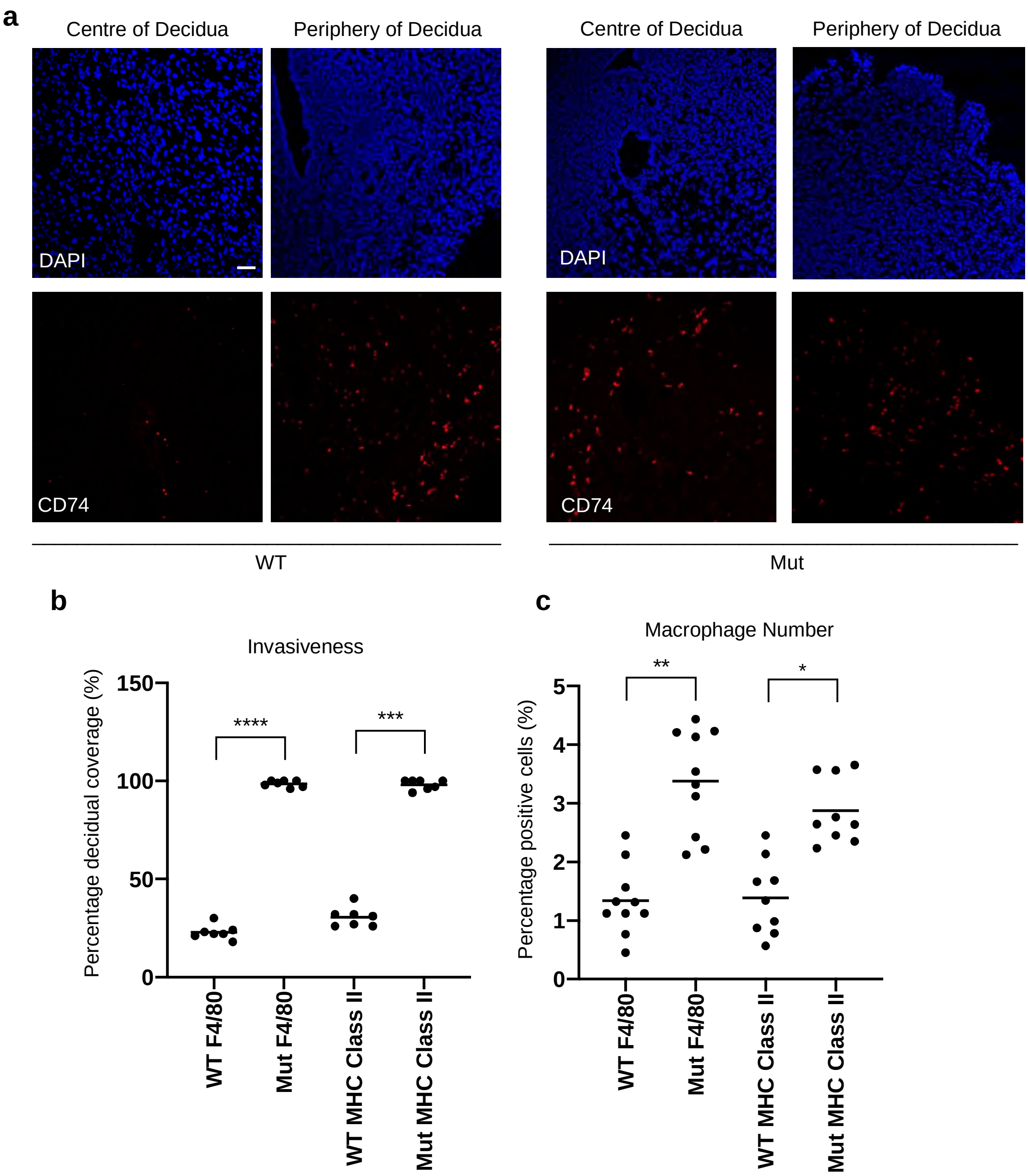

Supplementary Fig. 3. Increased macrophage invasion in E6.5 mutant decidua. Related to Fig. 6. **a**) IF staining of CD74 in E6.5 decidual regions in mutants. In contrast, staining in wild types is largely restricted to the surrounding myometrium (n=3 decidua per genotype). F4/80 and MHC Class II staining in E6.5 decidua identifies a significant increase in macrophage invasion in mutants as measured by (**b**) percentage area and (**c**) percentage of total cells. Individual data points from analysis of 7-10 tissue sections from 3 independent decidual samples per genotype are shown. Bars represent mean. Two-tailed unpaired Student's t-test $^{*}p = 8.62\times10^{-5}$, $^{**}p = 1.00\times10^{-5}$, $^{***}p=5.02\times10^{-13}$ and $^{****}p=2.73\times10^{-15}$. Source data are provided as a Source Data file. Scale bar = 100μm.

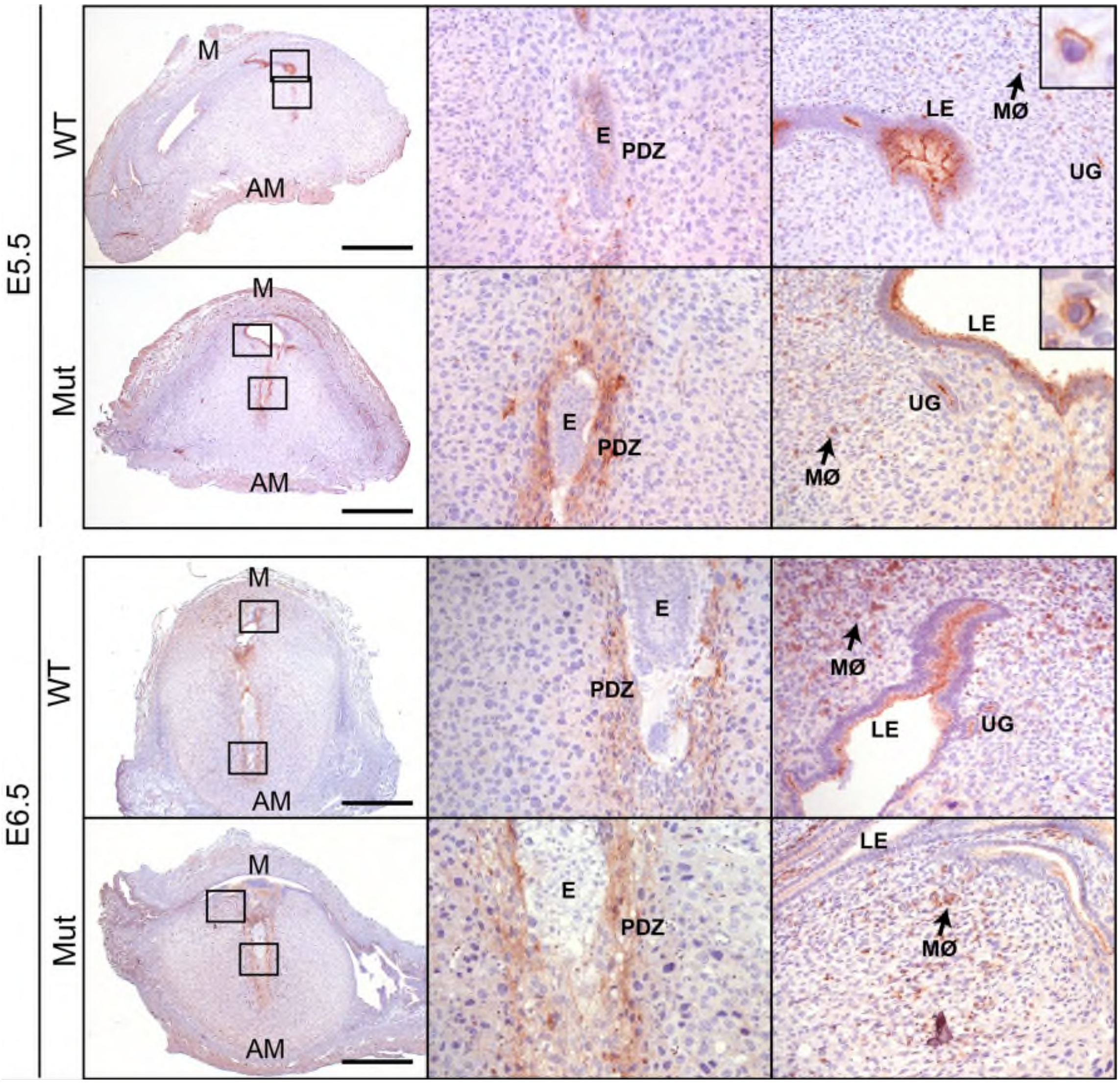

Supplementary Fig. 4. Immunohistochemical detection of Csf1 in wild type and Blimp1 mutant decidua at E5.5 and E6.5. Related to Fig. 6. Staining is observed on the plasma membrane of macrophages, on the apical surface of luminal and uterine glandular epithelial cells, and in PDZ stromal cells. Increased staining is observed in PDZ stromal cells in Blimp1 mutants. Results are representative of staining experiments using triplicate independent samples. WT = wild type, Mut = Blimp1 mutant, PDZ = primary decidual zone, M = mesometrial, AM = antimesometrial, E = embryo, MØ=macrophage, LE = luminal epithelium, UG = uterine gland. Scale bars = 1 mm.

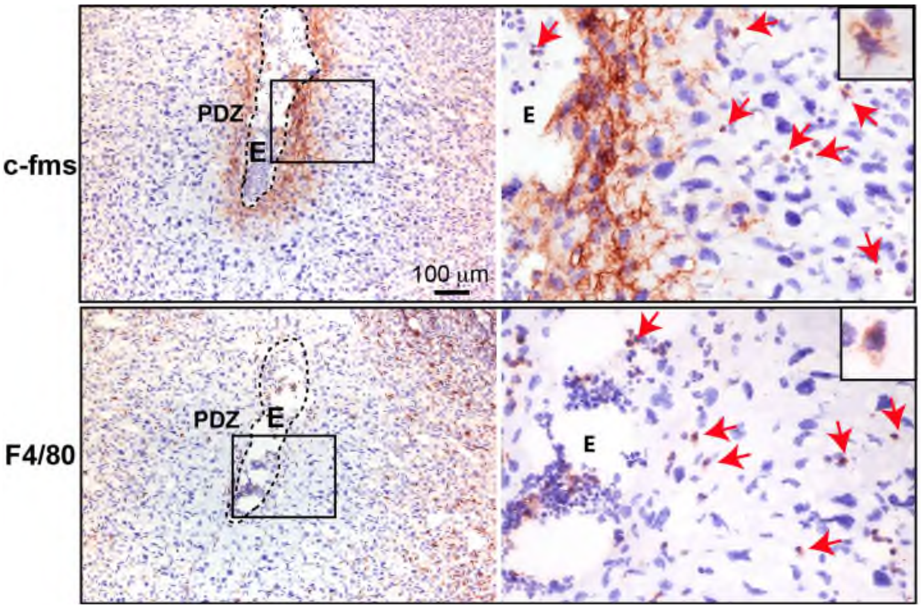

Supplementary Fig. 5. Immunohistochemical detection of c-fms and F4/80 in E6.5 Blimp1 mutant decidua. Related to Fig. 6d. In addition to strong staining in the PDZ, c-fms is detected on macrophages (red arrows) near the embryo (dotted line). F4/80 positive macrophages (red arrows) are present in the same region in serial sections (red arrows). Results are representative of staining experiments using triplicate independent samples. PDZ = primary decidual zone, E = embryo. Scale bars = 100 μm.

Antibody	Dilution	Source	Identifier
Rat monoclonal anti-Blimp1 clone 5E7	1/500	Santa Cruz	sc-130917
Rabbit polyclonal anti-Eomes	1/400	Abcam	ab23345
Rabbit polyclonal anti-Ki67	1/200	Abcam	ab15580
Rabbit polyclonal anti RFP	1/200	Rockland antibodies	600-401-379
Guinea-Pig polyclonal-K8	1/200	Progen Biotechnik	GP11
Rabbit polyclonal anti-Zo-1	1/200	Invitrogen	40-2200
Mouse monoclonal anti E-cadherin	1/200	BD Biosciences	610182
Rat monoclonal anti-mouse F4/80	1/200	Serotec	MCA497R
Rat monoclonal anti-MHC Class II	1/200	Abcam	ab139365
Rat monoclonal anti-CD74	1/200	Santa Cruz	sc-19627
Rat monoclonal anti-C-fms	1/500	Invitrogen	14-1152-81
Rabbit monoclonal anti-Csf1	1/500	Abcam	ab233387
Goat polyclonal anti-Oct4 -	1/200	Santa Cruz	sc-8628
Mouse monoclonal anti-BrdU	1/1000	Becton Dickinson	347580
Rabbit anti-rat IgG	1/400	Vector Laboratories	AI-4001
Donkey anti-rabbit Alexa Fluor 594	1/400	Invitrogen	A21207
Goat anti-guinea pig Alexa Fluor 488	1/400	Invitrogen	A11073
Donkey anti-mouse Alexa Fluor 488	1/400	Invitrogen	A21202
Donkey anti-rat Alexa Fluor 594	1/400	Molecular Probes	A21209
Donkey anti-goat Alexa Fluor 594	1/400	Invitrogen	A11058
Donkey anti-rabbit Alexa 488	1/400	Molecular Probes	A21206
Rabbit polyclonal anti-β-tubulin	1/1000	Santa Cruz	sc-9104
Donkey anti-rat Ig-HRP	1/2000	GE Healthcare	NA935
Donkey anti-rabbit Ig-HRP	1/2000	GE Healthcare	NA934

Supplementary Table 1. Antibodies used for immunostaining and Western blot analysis.

**Target**	**Forward Primer**	**Reverse Primer**	**Product size**
Csf1	ATGGACACCTGAAGGTCCTG	GTTAGCATTGGGGGTGTTGT	188bp
Ifnγ	*GAGGAACTGGCAAAAGGATG*	*TGAGCTCATTGAATGCTTGG*	*242*
Hprt	GCTGGTGAAAAGGACCTCT	CACAGGACTAGAACACCTGC	249bp

Supplementary Table 2. qPCR primers.
