# Supplementary material for: The transcriptional repressor Blimp1/PRDM1 regulates the maternal decidual response in mice
Source: Nat Commun. 2020 Jun 3;11:2782. doi: 10.1038/s41467-020-16603-z (PMC7270082; doi:10.1038/s41467-020-16603-z)
Supplement: Supplementary file 11 — Description of Additional Supplementary Files [file 41467_2020_16603_MOESM11_ESM.pdf]

Title: Supplementary Dataset 1.
Description: Related to Figure 4a. RNA-Seq analysis of E5.5 Blimp1 mutant versus wild type decidua. Differentially expressed genes (n=249 up-regulated and 252 down-regulated) based on DeSeq2 statistical significance with multiple testing correction (FDR cutoff of 0.05), ≥ 2 fold change in expression and FPKM of ≥ 1 (in all samples within at least one group). Analysis was performed using SeqMonk V1.45.4, genome build = mm10.

Title: Supplementary Dataset 2.
Description: Related to Figure 4a. RNA-Seq analysis of E6.5 Blimp1 mutant versus wild type decidua. Differentially expressed genes (n=703 up-regulated and 458 down-regulated) based on DeSeq2 statistical significance with multiple testing correction (FDR cutoff of 0.05), ≥ 2 fold change in expression and FPKM of ≥ 1 (in all samples within at least one group). Analysis performed using SeqMonk V1.45.4, genome build = mm10.

Title: Supplementary Dataset 3.
Description: Related to Figure 4b. Enriched GO terms among up- and down-regulated genes in E6.5 Blimp1 mutant decidua based on Biological Process with affinity propagation as performed using WebGestalt 2019 (http://webgestalt.org) with Benjamini-Hochberg multiple testing correction.

Title: Supplementary Dataset 4.
Description: Related to Figure 4e. IFNγ-responsive genes (n=134) based on GO:0034341, filtered for Mus musculus were identified using AMIGO2 (http://amigo.geneontology.org/amigo). DESeq2 FDR and fold change in E6.5 Blimp1 mutant relative to wild type decidua are indicated.

Title: Supplementary Dataset 5.
Description: Related to Figure 5a. Genome-wide Blimp1 binding sites in E6.5 decidua. High confidence peaks were identified by intersecting peak areas in triplicate GFP ChIP of NEG samples. Non-specific peaks were removed by subtracting overlapping peaks called in triplicate GFP ChIP of wild type samples. ChIP peak coordinates relate to the first replicate of the GFP antibody ChIP of NEG decidua. Overlapping peaks from E18.5 BEG mouse small intestine (Mould et al., 2015; https://doi.org/10.1371/journal.pgen.1005375) and other NEG mouse tissues (Mitani et al., 2017; https://doi.org/10.1093/nar/gkx798) are indicated. Gene/peak associations were identified using GREAT V3.0 (http://great.stanford.edu/public/html/) based on the single nearest gene option ± 100 kb of gene TSSs.

Title: Supplementary Dataset 6.
Description: Related to Figure 5b. Over-represented gene categories associated with common (n=1129) and decidual-specific Blimp1 ChIP peak subsets (n=2,962) within 100 kb of gene TSSs based on PANTHER pathway. Analysis was performed using GREAT V3.0 bionomial test with multiple testing correction, species assembly: mm10.

Title: Supplementary Dataset 7.
Description: Blimp1 target genes altered ≥ 2 fold in E6.5 Blimp1 mutant decidua as shown in Figure 5c.

Title: Supplementary Dataset 8.
Description: Enriched GO terms among up-regulated Blimp1 target genes in E6.5 Blimp1 mutant decidua as shown in Figure 5d, based on Biological Process with affinity propagation as performed using WebGestalt 2019 (http://webgestalt.org) with Benjamini-Hochberg multiple testing correction.
